# A Lightweight 1D-CNN-Transformer for Bearing Fault Diagnosis Under Limited Data and AWGN Interference

**DOI:** 10.3390/s26092574

**Published:** 2026-04-22

**Authors:** Yifan Guo, Yijie Zhi, Renyi Qi, Ming Cai

**Affiliations:** 1 ZJU-UIUC Institute, Zhejiang University, Haining 314400, China; 3230112158@zju.edu.cn (Y.G.); 3230111853@zju.edu.cn (R.Q.); 2 College of Computer Science and Technology, Zhejiang University, Hangzhou 310027, China; zhiyijie@zju.edu.cn

**Keywords:** fault diagnosis, bearing fault detection, deep learning, Transformer, condition monitoring, robust classification

## Abstract

Intelligent bearing fault diagnosis is essential for maintaining the reliability of rotating machinery. However, deploying deep learning models in industrial environments is often constrained by a lack of labeled data, environmental noise, and strict hardware limits. To address these connected challenges, this paper proposes 1D-CNN-Trans, a flexible and resource-efficient hybrid framework. Designed for supervised diagnosis with restricted data, the configurable model combines a compact one-dimensional convolutional neural network (1D-CNN) for local feature extraction, a Transformer encoder for capturing long-range temporal dependencies, and an optional squeeze-and-excitation (SE) module for channel recalibration under favorable conditions. The method is evaluated on two standard mechanical benchmarks under limited sample conditions, controlled additive white Gaussian noise (AWGN), and dynamic non-stationary interference. Experimental results indicate that 1D-CNN-Trans shows improved robustness under interference compared to selected baselines, notably improving accuracy against a standard CNN backbone. Furthermore, findings indicate that while the Transformer ensures noise robustness, channel recalibration (via SE) introduces optimization instability under extreme sparsity and noise. Consequently, we reposition the architecture as a configurable framework where recalibration is conditionally activated. Finally, theoretical complexity analysis is provided to validate the model’s low computational burden, indicating its general feasibility for resource-constrained scenarios.

## 1. Introduction

Rolling element bearings are critical components in rotating machinery. Their degradation or failure can lead to system breakdowns, economic losses, and safety hazards [1,2]. Data-driven fault diagnosis using deep learning, particularly convolutional neural networks (CNNs), provides a reliable approach to automated structural health monitoring [3,4,5]. Recent reviews further show that intelligent diagnosis research has expanded from conventional CNN pipelines toward transfer learning and Transformer-based hybrid architectures for rotating machinery and bearing monitoring [6,7,8].

Despite algorithmic progress, deploying these solutions in practical scenarios is often limited by engineering constraints. Recent studies increasingly treat low-latency diagnosis on resource-constrained hardware as a core requirement rather than a secondary implementation issue [9,10]. As the industry shifts toward edge computing, traditional diagnostic pipelines that transmit continuous high-frequency vibration data to centralized servers face challenges regarding communication latency and bandwidth consumption. Consequently, executing diagnosis directly on resource-constrained hardware has become a priority. However, highly accurate deep networks often possess millions of parameters, which may exceed the memory limits of embedded devices.

Compounding these deployment restrictions are limited annotated samples and environmental noise. Recent reviews on transfer learning and bearing diagnosis consistently note that industrial datasets are often imbalanced, weakly labeled, and distribution-shifted, making straightforward supervised training difficult [8,11,12]. Furthermore, vibration signals are often contaminated by measurement noise. Standard feed-forward CNNs, restricted by localized receptive fields, often struggle to isolate fault-induced transient impacts under low signal-to-noise ratio (SNR) conditions. Under limited labels, recent studies have therefore explored few-shot learning, noisy-label learning, and physics-based sample generation as complementary directions [13,14,15,16].

To address these challenges, this paper presents a configurable **1D-CNN-Trans** framework aimed at supervised diagnosis under limited data and controlled AWGN interference. Recent studies have shown the promise of lightweight CNN-Transformer hybrids for rotating machinery diagnosis under complex or noisy conditions [17,18,19,20,21], but a compact configuration tailored to limited supervised samples and controlled AWGN remains insufficiently explored. Instead of relying on deep architectures, we deploy a simplified 1D-CNN to extract local feature tokens. These tokens are then processed by a Transformer encoder to capture global dependencies across the sequence. Importantly, an SE module is provided as an optional extension to recalibrate feature channels, with the formal acknowledgment that such gating mechanisms can degrade stability under extreme data scarcity and noise. This research focuses on the engineering application of supervised diagnosis with limited labeled samples, rather than standard meta-learning defined few-shot scenarios.

The primary contributions of this paper are summarized as follows:

1. We propose a flexible hybrid framework integrating a simplified 1D-CNN and a Transformer encoder, designed as a configurable solution for bearing fault diagnosis under limited samples and AWGN conditions.
2. We demonstrate that while the Transformer consistently improves robustness, the SE channel recalibration negatively impacts optimization under extreme noise and data sparsity constraints, thereby establishing guidelines for condition-dependent module activation.
3. We evaluate the architecture’s configuring boundaries under restricted conditions (20 training samples per class and sub-zero SNRs) across the CWRU and JNU datasets. Theoretical complexity analysis is provided to validate the model’s low computational footprint.

The remainder of this paper is organized as follows: Section 2 reviews related literature. Section 3 elaborates on the proposed methodology. Section 4 provides experimental validations, ablation studies, and theoretical runtime estimations. Finally, Section 5 concludes the paper.

## 2. Related Work

### 2.1. Traditional CNNs and Noise Limitations

The transition from traditional machine learning to 1D-CNNs reduced manual feature engineering in fault diagnosis, allowing the extraction of shift-invariant transient features directly from raw temporal vibrations [3]. Recent reviews and raw-signal studies show that 1D convolution remains an effective backbone for bearing diagnosis because it preserves temporal locality while avoiding handcrafted time-frequency preprocessing in the simplest setting [7,22]. However, pure CNN architectures may experience performance degradation under strong noise. Because convolutional kernels possess restricted, localized receptive fields, they may treat high-amplitude random noise as valid high-frequency components. While deepening the CNN could improve robustness, it directly conflicts with the resource constraints required for practical edge deployment [9,10].

### 2.2. Transformers and Lightweight Adaptations

To transcend local receptive restrictions, Transformer architectures are increasingly considered for time-series diagnosis [6]. Originally introduced for sequence modeling [23] and later adapted for vision tasks [24], self-attention mechanisms help capture long-range temporal dependencies that are less accessible to purely local convolutional filters. Recent representative studies include CNN-enhanced time-series Transformers for multiple working conditions, adaptive lightweight 1D-XFormer models, and lightweight ConvFormer variants designed for small samples or heavy-noise settings [17,18,19,20]. In addition, anti-noise and deployable Transformer-based architectures have begun to emphasize the joint optimization of robustness and resource efficiency [21]. While progress has been made, developing a compact model optimized for the combined constraints of limited labeled samples and severe environmental noise remains an area requiring further engineering optimization.

### 2.3. Limited Data in Mechanical Fault Diagnosis

In real-world prognostics, acquiring well-balanced, comprehensive fault data is frequently challenging [8,11,12]. This has motivated various strategies to handle data scarcity, including Transformer-based few-shot learning under noisy labels, prototype-network formulations, physics-based sample generation, and joint collaborative adaptation for variable operating conditions [13,14,15,16]. In contrast to these methodologically heavy frameworks, this work focuses on a compact base architecture that can be effectively trained and deployed directly under data-scarce supervised conditions, avoiding the computational overhead of iterative episodic task sampling or generative modeling.

## 3. Methodology

### 3.1. Problem Formulation and Noise Model

Given a raw 1D vibration segment $x\in R^{L}$ with a discrete degradation label y∈{1,…,K}, the proposed network $f_{\theta }\left(\cdot \right)$ takes the corrupted signal $\tilde{x}$ and outputs logits $z=f_{\theta }\left(\tilde{x}\right)$. The predicted probabilities are computed via p=softmax(z). The diagnostic objective is to minimize the label-smoothed cross-entropy loss:

(1) $$L_{LS}=-\sum_{k=1}^{K}y_{k}^{ls}\log p_{k},$$

where $y_{k}^{ls}$ represents the smoothed label. The observed noisy signal is formulated as:

(2) $$\tilde{x}=x+n,\;n∼N\left(0,\sigma_{n}^{2}I\right).$$

This noise model represents controlled AWGN corruption for evaluation purposes and does not fully emulate complex industrial noise processes. For a targeted signal-to-noise ratio (SNR) in decibels, the noise variance is adjusted accordingly as:

(3) $$\sigma_{n}^{2}=\frac{P_{x}}{10^{{\mathrm{SNR}}_{\mathrm{dB}}/10}},\;P_{x}=\frac{1}{L}\sum_{t=1}^{L}x_{t}^{2}.$$

### 3.2. Overall Architecture

**Figure 1:**
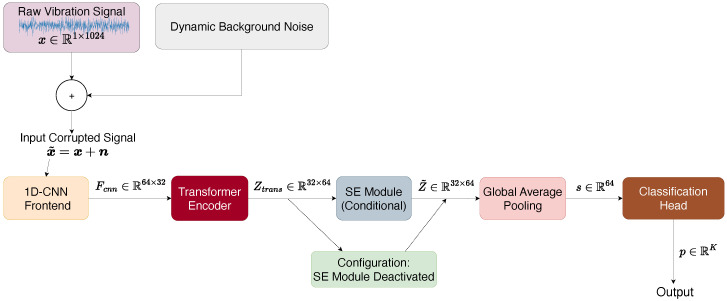
The overall architecture of the configurable 1D-CNN-Trans framework. The SE module incorporates a conditional bypass mechanism depending on the severity of the operational environment.

The 1D-CNN-Trans operates on 1D time-series inputs, and its overall architecture is illustrated in Figure 1. For an input window $\tilde{x}\in R^{1\times L}$ (where L=1024), the network first produces class logits through the composed feature extractor and classification head:

(4) $$z=\left(\phi_{cls}\circ \phi_{GAP}\circ \phi_{se}\circ \phi_{trans}\circ \phi_{cnn}\right)\left(\tilde{x}\right),$$

where $\phi_{cnn}$ represents the convolutional tokenizer, $\phi_{trans}$ is the Transformer encoder, $\phi_{se}$ acts as the channel recalibration function, $\phi_{GAP}$ applies Global Average Pooling across the temporal dimension, and $\phi_{cls}$ executes the final linear mapping to the *K* diagnostic categories. Prediction is then obtained by taking the argmax over the softmax probabilities:

(5) $$p=\mathrm{softmax}\left(z\right),\;\hat{y}=\underset{k\in \{1,\ldots ,K\}}{arg max}\;p_{k}.$$

### 3.3. Lightweight Tokenizer: 1D-CNN Frontend

We construct a simplified CNN frontend limited to two convolutional stages, which converts the input signal into a sequence of feature tokens. The first convolutional layer performs initial downsampling using a wide kernel ($K_{1}=64$) and large stride ($S_{1}=8$). The secondary block refines features with ($K_{2}=16,S_{2}=4$). Following batch normalization and ReLU activation, the signal transforms into a feature matrix $F_{cnn}\in R^{C\times N}$, reducing L=1024 to a sequence length of N=32 across C=64 embedding dimensions. This shallow convolutional tokenizer design is consistent with recent hybrid diagnosis models that retain local inductive bias before self-attention while controlling parameter growth [18,20]. To address details regarding layer configurations, the comprehensive sequence of layers and their corresponding output sizes are summarized in Table 1.

### 3.4. Global Sequence Modeling: Transformer Encoder

The tokenized matrices enter the Transformer encoder. Standard multi-head self-attention (MHSA) [23] is applied along with positional embeddings. This design follows recent Transformer-based diagnosis studies that use attention to model longer-range temporal interactions [6,18,21]. Specifically, the CNN tokenizer outputs $F_{cnn}\in R^{C\times N}$. After transposition, the token sequence is added to positional embeddings $PosEnc\in R^{N\times C}$, yielding the initial Transformer input $Z_{0}\in R^{N\times C}$:

(6) $$Z_{0}=F_{cnn}^{T}+PosEnc.$$

For each attention head *i*, Queries ($Q_{i}$), Keys ($K_{i}$), and Values ($V_{i}$) are computed via linear projections:

(7) $$Q_{i}=Z_{0}W_{i}^{Q},\;K_{i}=Z_{0}W_{i}^{K},\;V_{i}=Z_{0}W_{i}^{V},$$

where $W_{i}^{Q},W_{i}^{K},W_{i}^{V}\in R^{C\times d_{k}}$ and $d_{k}=d_{model}/h$. The scaled dot-product attention is defined as:

(8) $${\mathrm{head}}_{i}=\mathrm{softmax}\left(\frac{Q_{i}K_{i}^{⊤}}{\sqrt{d_{k}}}\right)V_{i}.$$

The outputs of the *h* heads are then concatenated and linearly projected via $W_{O}\in R^{hd_{k}\times C}$:

(9) $$\mathrm{MHSA}\left(Z_{0}\right)=\mathrm{Concat}\left({\mathrm{head}}_{1},\ldots ,{\mathrm{head}}_{h}\right)W_{O}.$$

The Transformer encoder is introduced to model longer-range temporal dependencies beyond local convolutional receptive fields. Although the convolutional tokenizer may induce correlations among neighboring tokens, the attention mechanism can still help aggregate informative temporal relationships across the sequence. Under the evaluated AWGN-corrupted settings, the observed performance gains are consistent with the hypothesis that the Transformer component improves robustness; however, this effect is not claimed as a formal theoretical guarantee. A possible interpretation is that recurrent fault-related temporal patterns can produce more stable cross-token correlations than random perturbations after projection, but this remains an empirical observation rather than a formal proof in the present work. The fused MHSA output $Z_{\mathrm{trans}}\in R^{N\times C}$ is then generated through feed-forward networks (FFNs) and layer normalization (LN). This architecture uses h=4 heads across 2 layers.

### 3.5. Channel Recalibration: SE Module

The output $Z_{\mathrm{trans}}\in R^{N\times C}$ subsequently passes through a squeeze-and-excitation (SE) module [25]. The SE block is used to adaptively recalibrate channel responses, whose effectiveness is further examined in the ablation study. Global channel descriptors $s\in R^{C}$ are generated by spatial pooling:

(10) $$s_{c}=\frac{1}{N}\sum_{t=1}^{N}Z_{\mathrm{trans}}\left(t,c\right),\;c=1,\ldots ,C.$$

This mechanism generates dynamic channel weights $g\in R^{C}$:

(11) $$g=\sigma \left(W_{2}\delta \left(W_{1}s\right)\right),$$

where $W_{1}\in R^{\frac{C}{r}\times C}$ and $W_{2}\in R^{C\times \frac{C}{r}}$ are the transform matrices of two fully connected layers parameterized by a dimensionality reduction ratio *r*, using ReLU (δ) and Sigmoid (σ) activations. These dynamic weights recalibrate the representation as $\tilde{Z}\left(t,c\right)=g_{c}\cdot Z_{\mathrm{trans}}\left(t,c\right)$ prior to global pooling.

## 4. Experimental Validations

### 4.1. Experimental Setup and Implementation Details

To evaluate the proposed network, experiments are conducted on two standard benchmarks: the Case Western Reserve University (CWRU) [26] and Jiangnan University (JNU) bearing datasets. For both datasets, data is segmented using a non-overlapping window of length L=1024 data points. To evaluate performance under constrained scenarios, exactly 20 samples per class are randomly selected for training, while the remaining available samples are used for testing. AWGN was added only to the test samples to evaluate robustness under different SNR conditions (ranging from 0 dB down to −12 dB), while the training samples remained unchanged. This precise evaluation protocol is purposefully designed to simulate a common industrial emergency: the model completes training in a controlled, clean environment but suddenly encounters unknown sensor degradation or environmental noise during actual deployment. Although real-world industrial environments often generate noisy training datasets—a challenge actively addressed by recent noisy-label and unsupervised learning literature—this decoupled evaluation specifically isolates and measures the network’s zero-shot resilience against post-deployment sensor degradation. Such a setup strictly tests the architecture’s intrinsic robustness against *unexpected interference*, avoiding data distribution leakage from the training phase.

**CWRU Dataset:** We structure a 10-class discrimination task containing normal operations against three varying severities (0.007, 0.014, and 0.021 inches) for inner race, outer race, and ball faults.

**JNU Dataset:** Recorded under distinct operational conditions from CWRU, we select the 1000 RPM condition to construct a 4-class fault domain (normal, inner race, outer race, ball fault).

**Training Specifications:** All evaluated internal baseline models were trained with the same optimizer, batch size, number of epochs, and learning-rate schedule to reduce training-related confounding factors. Architectural hyperparameters were kept comparable where possible, preserving each model’s intended structure. The models are optimized using the AdamW optimizer [27] over 80 epochs with an initial learning rate of $5\times 10^{-4}$, weight decay of $1\times 10^{-4}$, and a cosine annealing learning rate scheduler [28]. The batch size is set to 32, and label smoothing (α=0.1) [29] is applied. No separate validation set was used to perform early stopping because of the extremely limited number of labeled training samples (20 per class). However, empirical observation of the training logs across all independent random seeds indicated that the training loss consistently converged and stabilized prior to the fixed 80th epoch, mitigating the risk of capturing an unstable training state. To account for statistical variance, all experiments are independently evaluated across 10 distinct random seeds. For fair comparison, the 1D-XFormer external baseline was trained and evaluated using the exact same data partition, validation-free AWGN settings, optimizer configuration, training schedule, and 10-seed repetition protocol.

### 4.2. Baseline Models

To analyze the contribution of each component, four internal baseline models were considered:

- **1D-CNN:** The backbone utilizing simplified convolutional layers coupled with global average pooling.
- **1D-CNN-SE:** Identical to the foundational CNN, appended with an SE module.
- **1D-CNN-Trans-NoSE (Ablation Baseline):** Integrates the Transformer encoder but excludes the final SE gating.
- **1D-CNN-Trans (Ours):** The complete integrated architecture.

In addition, one recent lightweight Transformer-based diagnosis model, Adaptive Lightweight 1D-XFormer [17], was included as an external reference. We conservatively reimplemented this baseline following its core architectural description and evaluated it under the exact same data split, AWGN injection protocol, optimizer, and training schedule as the proposed method. This comparison provides a broader reference under a mathematically matched evaluation protocol.

### 4.3. Theoretical Complexity and Runtime Reference

Table 2 reports inference latency across desktop-class reference platforms and a physical Target Edge Device (TED). The proposed model contains 104,826 parameters and requires 3.67 M multiply-accumulate operations (MACs) per input window, indicating a compact architecture compared with deeper diagnosis models. To validate deployment feasibility beyond desktop hardware, the model was exported to the industry-standard ONNX format and benchmarked on a Raspberry Pi 4B (quad-core ARM Cortex-A72 at 1.5 GHz, 4 GB RAM), a representative resource-constrained edge platform. In the FP32 configuration utilizing all four cores, the model achieves a single-sample mean inference latency of 1.98 ms (throughput: 504 samples/s) and a peak batched throughput exceeding 1068 samples/s at batch size 8, with a runtime memory overhead (RSS delta) of approximately 8 MB. INT8 quantization reduced the ONNX model footprint from 463.6 KB to 419.7 KB (a 9.5% reduction); however, single-sample INT8 latency was marginally higher than FP32 (2.33 ms vs. 1.98 ms), attributable to the absence of hardware-native integer acceleration on the Cortex-A72 and the dequantization overhead incurred by the ONNX Runtime. Both model variants comfortably satisfy sub-megabyte storage constraints typical of edge deployments [9,10]. These results confirm that the proposed architecture achieves real-time, single-sample diagnosis on a physical edge device without architectural modification or specialized hardware acceleration.

### 4.4. Results and Discussion on CWRU Dataset

Table 3 details the diagnostic accuracy across varying SNR levels for the evaluated models. While Table 3 primarily reports top-1 classification accuracy for clarity, we independently evaluated the configurations using Precision, Recall, and F1-Score metrics to verify classification balance under noisy regimes. These supplementary metrics exhibited near-identical trends with the reported accuracy across all evaluated conditions. For example, at −8 dB, the foundational 1D-CNN exhibited severe classification decay (Precision = 56.2%, Recall = 55.4%, F1-Score = 55.6%), whereas the proposed 1D-CNN-Trans-NoSE successfully preserved boundary margins across balanced classes (Precision = 69.8%, Recall = 69.2%, F1-Score = 69.4%), thereby corroborating the reliability of the baseline accuracy metric under severe interference. Under noise-free conditions (SNR = None), both CNN and Transformer-based models performed exceptionally well, achieving above 99% mean accuracy.

**Figure 2:**
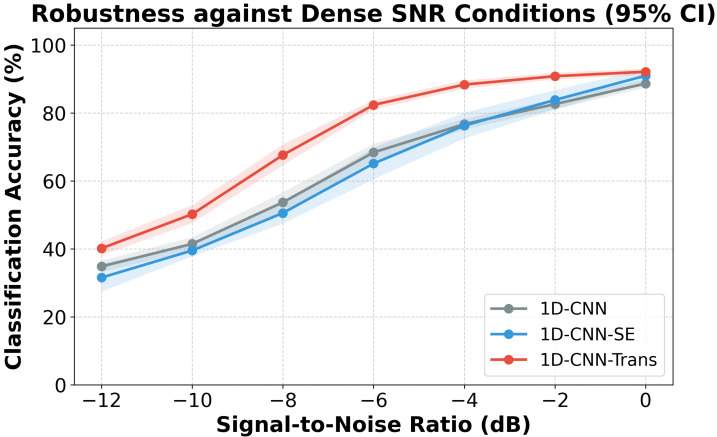
Classification accuracy with standard deviation bands over 10 independent runs across different AWGN conditions on CWRU.

The CWRU results are summarized in Table 3 and Figure 2. At an SNR of −8 dB, the baseline *1D-CNN* achieved 53.7% accuracy, whereas *1D-CNN-Trans-NoSE* reached 71.4%, corresponding to an increase of 17.7 percentage points. This result is consistent with the hypothesis that the Transformer component provides noticeable resilience under severe AWGN corruption. Such a tendency is broadly consistent with recent lightweight Transformer-based diagnosis studies that also emphasize robustness under noisy or complex conditions [17,19,21].

Critically, for the full *1D-CNN-Trans* model, the addition of the SE module negatively affected performance at this extreme noise level, dropping accuracy to 67.6% at −8 dB. This empirical finding forces a reassessment of the SE block’s utility: while theoretically powerful, channel recalibration introduces severe optimization instability when labeled samples are extremely sparse and heavily corrupted by noise. Therefore, rather than proposing a rigid architecture that mandates the SE module, we formulate 1D-CNN-Trans as a configurable framework. Under relatively benign conditions, the SE branch can be activated, but under extreme scarcity and noise, the bare *1D-CNN-Trans-NoSE* configuration is formally recommended as the superior and more robust baseline.

As additional external references, the reimplemented Adaptive Lightweight 1D-XFormer and a standard lightweight backbone (1D-MobileNetV2 [30]) were included to benchmark recent mainstream topologies. Under severe noise interference (−8 dB), the proposed 1D-CNN-Trans-NoSE achieved 71.4%, indicating more favorable resilience compared to the 1D-XFormer (66.1%) and 1D-MobileNetV2 (63.4%) baselines. At lower noise interference levels (−4 dB and 0 dB), all three lightweight models converged to comparable, highly competitive accuracies exceeding 85%. These results suggest that the proposed architectural arrangement provides a reliable and consistent diagnostic configuration without necessitating heavy parameter footprints.

#### Robustness Under Non-Stationary Noise

To better reflect realistic industrial environments where interference is rarely purely stationary Gaussian, we further evaluated the proposed model under dynamic, non-stationary noise conditions. Specifically, a sinusoidal amplitude envelope modulates the instantaneous SNR across the signal window. For a base SNR of ${\mathrm{SNR}}_{\mathrm{base}}$ (dB) and a modulation depth Δ=5 dB, the time-varying local SNR at sample index *t* is defined as:

(12) $$\mathrm{SNR}\left(t\right)={\mathrm{SNR}}_{\mathrm{base}}+\Delta \sin\left(\frac{2\pi t}{L}\right),\;t=0,1,\ldots ,L-1,$$

where *L* is the window length. The corresponding per-sample noise variance follows:

(13) $$\sigma_{n}^{2}\left(t\right)=\frac{P_{x}}{10^{\;\mathrm{SNR}(t)/10}},$$

with $P_{x}$ denoting the mean signal power as defined in Equation (3). Each noise sample is then drawn independently as $n\left(t\right)∼N(0,\sigma_{n}^{2}\left(t\right))$, producing a non-stationary interference whose amplitude oscillates between the equivalent of $\left({\mathrm{SNR}}_{\mathrm{base}}-5\right)$ dB and $\left({\mathrm{SNR}}_{\mathrm{base}}+5\right)$ dB over one full period.

**Figure 3:**
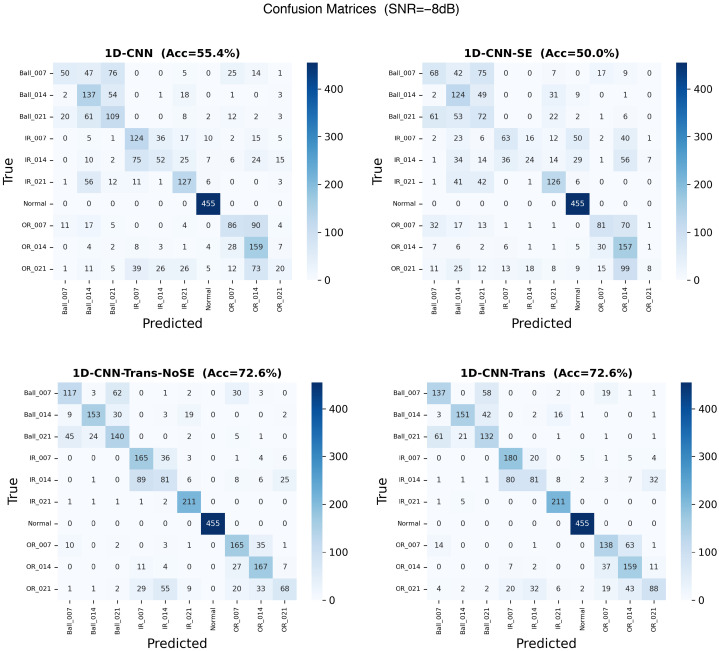
Confusion matrices demonstrating robust classification capabilities under rigorous dynamic, non-stationary noise (base SNR = −8 dB) across evaluated models.

Under a heavily corrupted base SNR of −8 dB (i.e., the instantaneous SNR sweeps from −13 dB to −3 dB), the foundational *1D-CNN* severely degraded to 49.88% mean accuracy. By contrast, the proposed *1D-CNN-Trans-NoSE* configuration retained an average accuracy of 69.22%, establishing an absolute improvement of over 19.3 percentage points. This finding empirically demonstrates that the global temporal aggregation provided by the Transformer encoder intrinsically resists time-varying, non-stationary industrial noise far better than purely localized convolutional filters. This is visually corroborated by the confusion matrices presented in Figure 3, wherein the Transformer-backed configuration successfully maintains stable diagnostic boundaries across adjacent fault severities despite the dynamic interference.

**Figure 4:**
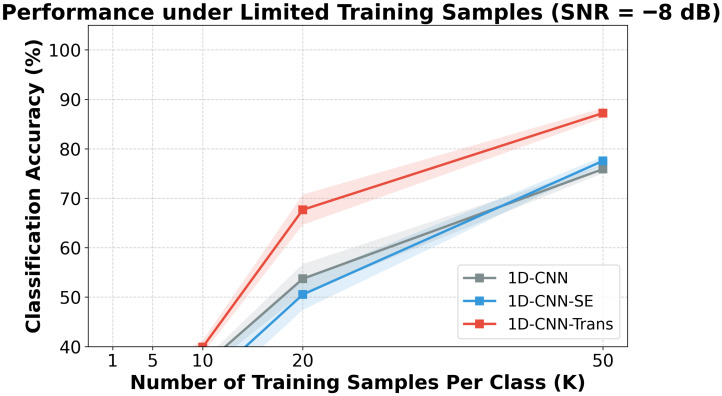
Accuracy under different numbers of training samples trained at a fixed test SNR = −8 dB on CWRU. The y-axis represents the evaluation accuracy on the test set (%).

Figure 4 explores performance under changing numbers of training samples subjected to an SNR of −8 dB. When restricted to only five training samples per class, the Transformer-based variants maintained a measurable distance from the CNN-only baselines. As sample volume increased, performance stabilized and improved across all models.

**Figure 5:**
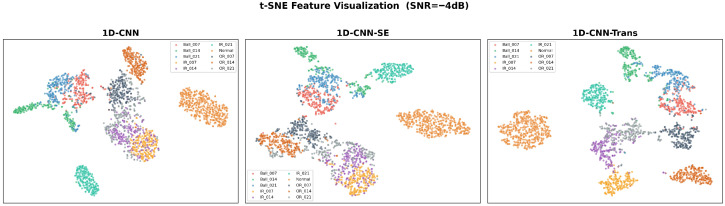
t-SNE visualization of embedding manifolds evaluated at SNR = −4 dB.

As a qualitative visualization, the t-SNE [31] plots in Figure 5 suggest that the Transformer-based features provide tighter class clustering than the CNN-only representations under the tested AWGN condition.

### 4.5. Generalization Validation on the JNU Dataset

Due to the revision scope and computational budget, the external lightweight Transformer comparison was conducted primarily on the CWRU benchmark, while the JNU experiments were utilized for validating the internal ablation mechanisms. The JNU results are presented in Table 4.

The JNU results align with those observed on the CWRU dataset, indicating consistent performance across different equipment configurations. At −8 dB, the *1D-CNN* achieved 55.7% accuracy, while the *1D-CNN-Trans-NoSE* raised the rate to 64.6%. The contribution of the SE block, achieving 64.3% accuracy in the full model, was again found to be condition-dependent. This supports the observation that channel recalibration may not always provide consistent gains when labeled data is extremely limited.

### 4.6. Limitations of the Current Study

Several limitations of this study should be acknowledged. First, the robustness assessment was restricted to artificial AWGN, which does not fully capture the complexity of real-world industrial noise; recent work has begun to examine varying-noise or stronger anti-noise settings with more specialized Transformer-based designs [21]. Second, the experiments were exclusively based on two public benchmark datasets; validation on data from actual industrial equipment is necessary. Third, although one recent lightweight Transformer baseline was incorporated, broader comparisons with additional few-shot, noisy-label, and sample-generation methods would further strengthen the reference baseline [13,14,15,16]. Finally, although deployment on a Raspberry Pi 4B has validated real-time inference feasibility on a representative edge platform, broader hardware coverage—particularly MCU-class targets with stricter memory hierarchies—remains necessary. Similarly, end-to-end system integration encompassing data acquisition, on-device inference, and automated alert dispatch within a live industrial environment represents a key direction for future work [10].

## 5. Conclusions

This paper presents a configurable 1D-CNN-Transformer framework adapted for bearing fault diagnosis under limited-data settings and controlled AWGN interference. By combining local convolutional feature extraction with Transformer-based temporal modeling, the framework demonstrates improved accuracy over CNN baselines across the evaluated conditions on both the CWRU and JNU datasets. Our structural ablation reveals a critical dynamic: while the Transformer encoder reliably establishes noise robustness, the SE module actively impairs optimization under extreme data sparsity and severe noise (e.g., −8 dB). Consequently, we reposition the architecture not as a monolithic model but as a flexible framework where the SE module is conditionally bypassed in high-noise, few-shot environments in favor of the resilient NoSE baseline. Edge deployment experiments on a Raspberry Pi 4B confirm real-time inference capability with single-sample latency under 2 ms and a sub-500 KB model footprint, substantiating the architecture’s applicability to resource-constrained platforms. Future work will extend hardware validation to MCU-class targets and evaluate robustness under unconstrained industrial noise distributions [10,17,21].

## Figures and Tables

**Table 1:** Detailed architecture of the 1D-CNN-Trans with corresponding output sizes.

Layer/Block	Configuration Details	Output Size (C×L)
Input Signal	Raw 1D vibration segment	1×1024
Conv1D Block 1	$K_{1}=64,S_{1}=8$, Ch =32, BN, ReLU	32×128
Conv1D Block 2	$K_{2}=16,S_{2}=4$, Ch =64, BN, ReLU	64×32
Transformer Encoder	2 Layers, 4 Heads, $d_{\mathrm{model}}=64$, FFN = 128	64×32
SE Module	Reduction ratio r=4, FC layers (Optional)	64×32
Global Avg Pool	Pool across temporal dimension	64×1
Classifier	Linear (64→K classes)	K×1

**Table 2:** Inference latency on desktop-class reference platforms and the target edge device (Raspberry Pi 4B).

Device/Environment	Precision	Batch Size	Latency (ms)	Throughput (SPS)
*Desktop Reference Platforms*				
macOS CPU (Apple M4)	FP32	1	0.3389	2951.1
macOS CPU (Apple M4)	FP32	16	2.7679	5780.5
macOS MPS (Apple M4)	FP32	16	0.8471	18,888.0
Windows CPU (Intel i7-13620H)	FP32	1	1.0429	958.8
*Target Edge Device*				
Raspberry Pi 4B (Cortex-A72)	FP32	1	1.9828	504.3
Raspberry Pi 4B (Cortex-A72)	FP32	8	7.4892	1068.2
Raspberry Pi 4B (Cortex-A72)	INT8	1	2.3272	429.7

**Table 3:** Classification accuracy (%) reported as mean ± standard deviation over 10 random seeds on CWRU.

Model	−12 dB	−10 dB	−8 dB	−6 dB	−4 dB	−2 dB	0 dB
1D-CNN	34.8 ± 2.4	41.5 ± 2.7	53.7 ± 4.2	68.4 ± 3.5	76.7 ± 2.1	82.6 ± 2.2	88.6 ± 1.7
1D-CNN-SE	31.5 ± 5.8	39.5 ± 2.4	50.5 ± 4.4	65.1 ± 6.5	76.3 ± 5.3	83.8 ± 4.0	91.0 ± 2.7
Adaptive Lightweight 1D-XFormer	-	-	66.1 ± 2.6	-	86.2 ± 1.4	-	92.3 ± 1.0
1D-MobileNetV2 (External)	-	-	63.4 ± 5.1	-	87.6 ± 1.9	-	92.3 ± 1.1
1D-CNN-Trans-NoSE	42.4 ± 1.2	53.1 ± 3.2	71.4 ± 2.2	83.5 ± 1.3	88.6 ± 1.1	90.7 ± 1.1	91.6 ± 1.2
**1D-CNN-Trans (Ours)**	**40.1 ± 2.1**	**50.2 ± 2.6**	**67.6 ± 3.1**	**82.4 ± 1.4**	**88.3 ± 1.2**	**90.8 ± 1.0**	**92.1 ± 1.0**

**Table 4:** Classification accuracy (%) reported as mean ± standard deviation over 10 random seeds on the JNU dataset.

Model	−12 dB	−8 dB	−4 dB	0 dB	Params
1D-CNN	56.6 ± 1.1	55.7 ± 1.0	60.8 ± 1.8	68.5 ± 0.1	35,364
1D-CNN-SE	51.3 ± 4.7	52.2 ± 4.5	59.5 ± 4.2	66.1 ± 3.1	37,492
1D-CNN-Trans-NoSE	61.0 ± 0.2	64.6 ± 1.5	72.3 ± 0.8	74.8 ± 0.6	102,308
**1D-CNN-Trans (Ours)**	**60.6 ± 0.1**	**64.3 ± 0.6**	**71.5 ± 0.6**	**74.2 ± 0.3**	104,436

## Data Availability

Publicly available datasets were analyzed in this study. The Case Western Reserve University (CWRU) dataset can be downloaded from its official repository (https://engineering.case.edu/bearingdatacenter/download-data-file, accessed on 15 March 2026). The Jiangnan University (JNU) dataset is available via GitHub (https://github.com/ClarkGableWang/JNU-Bearing-Dataset, accessed on 15 March 2026).
